# Waking Salivary Cortisone vs Serum Cortisol in the Short Synacthen Test in Screening for Adrenocortical Insufficiency: Results of a Service Evaluation

**DOI:** 10.1111/cen.15279

**Published:** 2025-05-27

**Authors:** Adrian Heald, Mathilde Mordaunt, Natalie Gallant, Hannah Baker, Waseem Majeed, Rupinder Kochhar, Akheel A. Syed, Rajshekhar Mudaliar, Ramadan Abushufa, Fahmy Hanna, David Marshall, Ian Laing, Brian Keevil, Anthony A. Fryer

**Affiliations:** ^1^ University of Manchester Manchester UK; ^2^ Salford Royal Hospital Salford UK; ^3^ Tripoli University Tripoli Libya; ^4^ University Hospital North Midlands Stoke on‐Trent UK; ^5^ Royal Preston Hospital Fulwood UK; ^6^ Keele University Keele UK

**Keywords:** adrenal, insufficiency, salivary cortisone, short synacthen, testing

To the Editor,

Adrenocortical insufficiency is a potentially life‐threatening endocrine condition [1]. Irrespective of the cause, adrenal insufficiency is generally diagnosed by measuring morning serum cortisol followed by measurement of the acute response at 30 and sometimes also at 60 min following injection of synthetic corticotropin (ACTH[1‐24]) in the short synacthen test (SST) [2]. We previously described that in the United Kingdom a larger proportion of these tests are not strictly necessary because in most centres the results do not support the diagnosis of adrenocortical insufficiency [3, 4]. We here evaluated whether saliva cortisol/cortisone measurements can be applied in every day clinical practice (minimal chance of cross reaction with prescribed glucocorticoids) as an alternative to the SST (at least 92,000 done each year in England) to evaluate adrenocortical function [5].

A morning serum cortisol level can potentially also be used to screen for adrenocortical insufficiency [6, 7] followed by a SST if results are indeterminant. Waking salivary cortisone has been proposed as the first line screening test for adrenocortical insufficiency [6]. Salivary glucocorticoids are stable at room temperature, which means that the saliva sample mailed to the laboratory or dropped off next working day if necessary [8]. Furthermore, the sampling technique is very straightforward.

The landmark Debono study analysis [9] of waking salivary cortisone provided information similar to that of a SST in 70% of participants. Notably 83% of patients preferred home salivary sample collection to clinic attendance.

There are high levels of 11b‐hydroxysteroid dehydrogenase‐2 in the salivary gland [10]. This converts free cortisol to cortisone. Hence salivary cortisone correlates better with serum cortisol than salivary cortisol. Salivary cortisone levels are higher than salivary cortisol and consequently are detectable at low serum cortisol levels [11]. Similarly, Anderson et al. [12] through their study suggested that the salivary cortisone awakening response may be closely related to serum cortisol dynamics then salivary cortisol and thus an alternative marker for monitoring the HPA‐axis awakening response.

A question remains as to whether saliva cortisone measurements can be applied in every day clinical practice as an alternative to the SST. The purpose of this study was to describe how the new approach of evaluation of salivary cortisone can be applied in the real world clinical setting.

We undertook a service evaluation of salivary cortisone vs serum cortisol at baseline; 30 min and 60 min in the SST (as per the usual protocol at the centre). Patients undergoing a SST, provided a waking saliva simple for measurement of salivary cortisone which was sent to the clinical biochemistry laboratory when they attended for the SST.

Patients were recruited by consecutive sampling between 5 September 2024 and 16 December 2024. All patients were given written instructions on how to collect their saliva sample. Patients taking glucocorticoids were asked to omit these medicines the evening before and the day of the test until all samples were collected.

Salivary cortisone was measured by electrospray positive liquid chromatography tandem mass spectrometry [13, 14]. The cut‐offs applied were the the same as described by De Bono et al. [9]. We also used exactly the same immunoassay and LC‐MS/MS as described in that publication.

Serum cortisol was analysed by Immunoassay (Elecsys Cortisol II assay from Roche) according to the manufacturer's instructions. Lower limit of detection and quantification were 1.5 nmol/L and 3.0 nmol/L respectively.

Cortisone was extracted from saliva samples using supported liquid extraction. The extracted samples were measured by electrospray ionisation in positive ion mode using a Waters TQ‐XS mass spectrometer with an Acquity sample injector. Inter assay imprecision was < 5% across a range of concentrations from 5.0 to 150 nmol/L. Limit of detection and quantification were 0.1 and 0.3 nmol/L respectively.

Formal ethical permission was not required as this was a service evaluation of a new laboratory technique in comparison with existing practice [15].

Comparisons were made between waking salivary cortisone and serum cortisol at basal serum cortisol and at 30 and 60 min post Synacthen. Serum cortisol at 30 and 60 min post synacthen were dichotomised into those with values of < 450 nmol/L (fail) and those ≥ 450 nmol/L (pass). Salivary cortisone was categorised into fail (< 7 nmol/L), equivocal (7 to 16.9 nmol/L) and pass (≥ 17.0 nmol/L). Chi‐squared tested were used to compare distributions between groups. Salivary cortisone was then dichotomised into fail/equivocal < 17 nmol/L and pass (≥ 17 nmol/L) as a potential rule‐out tool. Kappa statistics were used to assess agreement between dichotomised parameters. Sensitivity, specificity plus positive and negative predictive values were calculated from 2 × 2 tables with post synacthen serum cortisol being used to define the ‘disease’ group. All analyses were conducted using STATA version 18, StataCorp, Texas, US.

We compared salivary cortisone with the SST evaluation of adrenocortical reserve in 27 individuals comprising 6 men (mean age 46.3 years) and 21 women (mean age 46.0 years). Mean BMI for men was 34.5 kg/m^2^ and for women was 28.7 kg/m^2^. The reasons for the tests were as follows: weaning off glucocorticoids in 6 patients, low serum cortisol in 7, fatigue in 4, possible hypopituitarism in 7, postoperatively following adrenalectomy for Cushing's Syndrome in 1, and autoimmune spectrum disorder in 2 patients.

Overall 70.4% of SST (19/27) indicated adequate adrenocortical function on the basis of 30 or 60 min serum cortisol, which is the criterion used at our centre.

For evaluation of waking salivary cortisone vs 30 min post synacthen serum cortisol of 450 nmol/in the 25 consecutive cases (Table 1) (in 2 cases there was only baseline cortisol and 60 post synacthen cortisol) there was 80% concordance between waking low salivary cortisone (< 7 nmol/L) and SST in terms of ‘fail’ with no false positives on the salivary cortisone (Figure 1). In terms of ‘pass’ 100% of waking salivary cortisone measurements (≥ 17 nmol/L) were also a pass on the SST. For ‘borderline’ salivary cortisone (7 to 16.9 nmol/L) 4/7 failed the SST and 3/7 passed. As a first line screening test all borderline salivary cortisone results would be further investigated with a SST.

**Table 1:** Waking salivary cortisone vs post synacthen cortisol at 30 min.

	SST serum cortisol (30 min)		
Salivary cortisone	< 450 nmol/L (fail)	≥ 450 nmol/L (pass)	Total
< 7 nmol/L (fail)	4 (80.0%)	1 (20.0%)	5
7–16.9 nmol/L (equivocal)	4 (57.1%)	3 (42.9%)	7
≥ 17 nmol/L (pass)	0 (0.0%)	13 (100.0%)	13
Total	8 (32.0%)	17 (68.0%)	25

**Figure 1:**
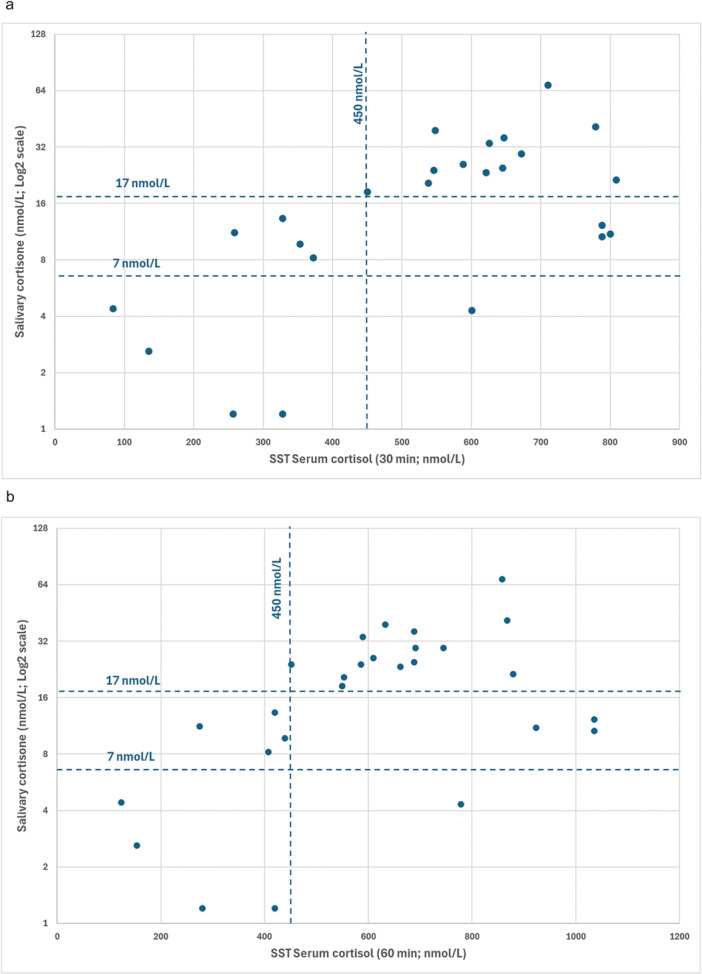
(a): Post synacthen cortisol at 30 min vs waking salivary cortisone. The patient in the right lower quadrant was taking Rigevidon combined oral contraceptive preparation at the time of the SST. (b): Post synacthen cortisol at 60 min vs waking salivary cortisone The patient in the right lower quadrant was taking Rigevidon combined oral contraceptive preparation at the time of the SST.

Overall agreement between SST 30 min serum cortisol value (< 450 vs ≥ 450 nmol/L) and salivary cortisone (< 17 vs ≥ 17 nmol/L) was 84% (kappa = 0.68, *p* < 0.001). In relation to diagnosis of adrenocortical insufficiency on the basis of the SST, sensitivity of salivary cortisone was 100% and specificity was 76.5%. Positive predictive value was 66.7% and negative predictive value was 100%.

Of those that passed the SST on the basis of the 30 min cortisol, 76.5% also passed on the basis of salivary cortisone.

For evaluation of waking salivary cortisone vs 60 min post synacthen serum cortisol of 450 nmol/L, in the 27 consecutive cases (Table 2) there was 80% concordance between waking low salivary cortisone (< 7nmol/L) and SST in terms of ‘fail’ with no false positives on the salivary cortisone (Figure 1b). In terms of ‘pass’ 100% of waking salivary cortisone measurements (≥ 17 nmol/L) were also a pass on the SST. For ‘borderline’ salivary cortisone (7–16.9 nmol/L) 4/7 failed the SST and 3/7 passed. As a first line screening test all borderline salivary cortisone results would be further investigated with SST.

**Table 2:** Waking salivary cortisone vs post synacthen cortisol at 60 min.

	SST serum cortisol (60 min)		
Salivary cortisone	< 450 nmol/L (fail)	≥ 450 nmol/L (pass)	Total
< 7 nmol/L (fail)	4 (80.0%)	1 (20.0%)	5
7–16.9 nmol/L (equivocal)	4 (57.1%)	3 (42.9%)	7
≥ 17 nmol/L (pass)	0 (0.0%)	15 (100.0%)	15
Total	8 (29.6%)	19 (70.4%)	27

Overall agreement between SST 60 min serum cortisol value (< 450 vs ≥ 450 nmol/L) and salivary cortisone (< 17 vs ≥ 17 nmol/L) was 85.2% (kappa = 0.69, *p* < 0.001). In relation to diagnosis of adrenal insufficiency on the basis of the SST, sensitivity was 100% and specificity was 78.9%. Positive predictive value was 66.7% and negative predictive value was 100%. Of those that passed the SST on the basis of the 60 min cortisol, 78.9% also passed on the basis of salivary cortisone.

The only patient in the right lower quadrant of both Figure 1a and Figure 1b where the discrepancy was greatest between salivary cortisone and serum cortisol in the SST was taking Rigevidon combined oral contraceptive preparation (COCP) at the time of the SST.

We also compared waking salivary cortisone with baseline 9am cortisol at a cut off of < 200 nmol/L to describe potential adrenocortical insufficiency, sensitivity of salivary cortisone was 80.0%, specificity 73.1%, positive predictive value 53.3%, negative predictive value 90.5%. Positive predictive value was 53.3% and negative predictive value 90.5%.

In summary, waking salivary cortisone did not falsely categorise anyone as having normal adrenocortical function. Of those that passed the SST more than 75% also passed on the basis of salivary cortisone whether the 30 min or 60 min post synacthen cortisol was used to define ‘a pass’.

We and others have shown that the majority of people undergoing an SST actually have normal adrenocortical function. We previously reported that out of 225 consecutive SST, 81.3% were a ‘pass’ on the basis of 30 or 60 min cortisol [3]. Eng et al. in a retrospective study investigating all SSTs performed over a year in a tertiary centre, found that of the 781 SSTs performed, 83.9% of SSTs showed an adequate cortisol response [16].

An advantage of salivary cortisone measurement is that the level of salivary cortisone is not influenced by the levels of cortisol binding globulin, so estrogen containing preparations in the form of the combined oral contraceptive pill (COCP) and hormone replacement treatment do not have to stopped 1 month before the test [17, 18]. This means that the likelihood of a falsely reassuring assessment of adrenocortical status is much less. This phenomenon is exemplified by Patient 9 whose salivary cortisone was low but whose 30 and 60 min serum cortisol levels were respectively 601 and 779 nmol/L (in the right lower quadrant of Figures 1a and 1b) who was taking Rigevidon COCP at the time of the SST, likely resulting in high levels of cortisol binding globulin and consequently giving misleadingly high levels of 30 and 60 min cortisol in the SST.

When compared with 9am cortisol at a cut off of < 200 nmol/L to describe adrenocortical insufficiency, performance against 0900 cortisol was less good with a lower negative predictive value of 90.5% vs 100% when compared with 0 or 60 min post synacthen cortisol. However it is the short synacthen test non 0900 serum cortisol that is the standard against which any other screening tests should be compared.

From a health economic point of view there are potential savings ‐ the cost of a SST is around £400 per test [19] compared with salivary cortisone at approximately £18 per sample including transport the reference laboratory. From a practical point of view postage of saliva samples is allowed through the regular mail in the UK as in many other countries [20]. Furthermore provision of a sample for salivary cortisone does not require venepuncture or attendance at hospital nor is there any cross reaction with prescribed glucocorticoids.

In relation to limitations, we accept that the number of cases reported here is relatively low. However, all cases were consecutive and we have been able to provide relevant clinical details on all of them. This was a pragmatic evaluation in a real world clinical setting.

It is clear from this service evaluation and the published research that the use of salivary cortisone as an assay for screening and diagnosing adrenocortical insufficiency using liquid chromatography tandem mass spectrometry, has potential for saving money, saving the patient a trip to hospital and mitigating the need for venepuncture as the first line test in screening for adrenocortical insufficiency with the caveat that there access to a laboratory offering the salivary cortisone assay is required.

In conclusion, waking salivary cortisone did not falsely categorise anyone as having normal adrenocortical function. Of those that passed the SST on the 60 min cortisol, 78.9% also passed on the basis of salivary cortisone and 76.5% who passed on the SST 30 min cortisol also passed on salivary cortisone.

We suggest that waking salivary cortisone could therefore be used as a safe alternative 1st line screening test which does not require venepuncture or attendance at hospital.

## Author Contributions

Mathilde Mordaunt, Hannah Baker and Adrian Heald wrote the manuscript with assistance from Natalie Gallant and data analysis by Anthony A. Fryer. Waseem Majeed, Rupinder Kochhar, Ramadan Abushufa, David Marshall, Akheel A. Syed and Anthony A. Fryer contributed to and have approved the final version of the manuscript. Brian Keevil, Rajshekhar Mudaliar, Fahmy Hanna and Ian Laing provided essential insights and senior review as did Anthony A. Fryer.

## Conflicts of Interest

The authors declare no conflicts of interest.

## Data Availability

The data that supports the findings of the study are available on reasonable request.

## References

[1] H. Raff and C. D. Zhang , “A New Approach ‐ Home Waking Salivary Cortisone to Screen for Adrenal Insufficiency,” NEJM Evidence 2, no. 2 (February 2023): EVIDe2200306. 38320042 10.1056/EVIDe2200306

[2] https://cks.nice.org.uk/topics/addisons-disease/diagnosis/confirming-the-diagnosis/, accessed 30 January 2025.

[3] M. Michaelidou , G. Yadegarfar , L. Morris , et al., “Recalibration of Thinking About Adrenocortical Function Assessment: How the ‘Random’ Cortisol Relates to the Short Synacthen Test Results,” Cardiovascular Endocrinology & Metabolism 10, no. 2 (April 2021): 137–145. 34113799 10.1097/XCE.0000000000000250 PMC8186517

[4] M. Michaelidou , G. Yadegarfar , L. Morris , et al., “What Is the Value of the 60‐minute Cortisol Measurement in the Short Synacthen Test (SST)? Evidence for the Defence,” International Journal of Clinical Practice 75, no. 8 (August 2021): e14417. 34289642 10.1111/ijcp.14417

[5] K. K. Chatha , J. G. Middle , and E. S. Kilpatrick , “National UK Audit of the Short Synacthen Test,” Annals of Clinical Biochemistry: International Journal of Laboratory Medicine 47, no. Pt 2 (March 2010): 158–164. 10.1258/acb.2009.009209 20150215

[6] M. Debono , C. Ghobadi , A. Rostami‐Hodjegan , et al., “Modifiedrelease Hydrocortisone to Provide Circadian Cortisol Profiles,” Journal of Clinical Endocrinology & Metabolism 94 (2009): 1548–1554. 19223520 10.1210/jc.2008-2380 PMC2684472

[7] C. P. Woods , N. Argese , M. Chapman , et al., “Adrenal Suppression in Patients Taking Inhaled Glucocorticoids Is Highly Prevalent and Management Can be Guided by Morning Cortisol,” European Journal of Endocrinology 173 (2015): 633–642. 26294794 10.1530/EJE-15-0608 PMC4588051

[8] A. A. Nalla , G. Thomsen , G. M. Knudsen , and V. G. Frokjaer , “The Effect of Storage Conditions on Salivary Cortisol Concentrations Using an Enzyme Immunoassay,” Scandinavian Journal of Clinical and Laboratory Investigation 75 (2015): 92–95. 25510953 10.3109/00365513.2014.985252

[9] M. Debono , C. J. Elder , J. Lewis , et al., “Home Waking Salivary Cortisone to Screen for Adrenal Insufficiency,” NEJM Evidence 2, no. 2 (February 2023): EVIDoa2200182. 38320034 10.1056/EVIDoa2200182

[10] M. Debono , R. F. Harrison , M. J. Whitaker , et al., “Salivary Cortisone Reflects Cortisol Exposure Under Physiological Conditions and After Hydrocortisone,” Journal of Clinical Endocrinology & Metabolism 101 (2016): 1469–1477. 26812690 10.1210/jc.2015-3694

[11] I. Perogamvros , B. G. Keevil , D. W. Ray , and P. J. Trainer , “Salivary Cortisone Is a Potential Biomarker for Serum Free Cortisol,” Journal of Clinical Endocrinology & Metabolism 95, no. 11 (November 2010): 4951–4958, 10.1210/jc.2010-1215. 20685855

[12] T. Anderson and L. Wideman , “The Association Between the Cortisol and Cortisone Awakening Responses,” Psychoneuroendocrinology 152 (2023): 106075, 10.1016/j.psyneuen.2023.106075. 36933271

[13] C. S. Ho , C. W. Lam , M. H. Chan , et al., “Electrospray Ionisation Mass Spectrometry: Principles and Clinical Applications,” Clinical Biochemist Reviews 24 (2003): 3–12. 18568044 PMC1853331

[14] R. L. Jones , L. J. Owen , J. E. Adaway , and B. G. Keevil , “Simultaneous Analysis of Cortisol and Cortisone in Saliva Using XLC‐MS/MS for Fully Automated Online Solid Phase Extraction,” Journal of Chromatography B 881–882 (January 2012): 42–48, 10.1016/j.jchromb.2011.11.036. 22178191

[15] https://www.hra-decisiontools.org.uk/ethics, accessed 13 December 2024.

[16] P. C. Eng , V. Ramadoss , L. Y. L. Tan , L. Z. Ong , D. S. Deepak , and C. M. Khoo , “Investigating the Clinical Appropriateness of Short Synacthen Testing and Utility of Pretest Cortisol to Predict Short Synacthen Testing Outcomes: A Tertiary Center Experience in Southeast Asia,” Endocrine Practice 31, no. 1 (January 2025): 34–41. 39428067 10.1016/j.eprac.2024.10.006

[17] N. Bäcklund , S. Lundstedt , A. Tornevi , et al., “Salivary Cortisol and Cortisone can Circumvent Confounding Effects of Oral Contraceptives in the Short Synacthen Test,” Journal of Clinical Endocrinology & Metabolism 109, no. 7 (June 2024): 1899–1906. 38173358 10.1210/clinem/dgad763 PMC11180507

[18] N. Bäcklund , S. Lundstedt , A. Tornevi , et al., “Salivary Cortisol and Cortisone can Circumvent Confounding Effects of Oral Contraceptives in the Short Synacthen Test,” Journal of Clinical Endocrinology and Metabolism 109, no. 7 (July 2024): 1899–1906. 38173358 10.1210/clinem/dgad763 PMC11180507

[19] https://www.nice.org.uk/guidance/ng243/evidence/d-diagnostic-tests-and-diagnostic-thresholds-for-referral-pdf-13494270784, accessed 20 January 2025.

[20] J. Blair , J. Adaway , B. Keevil , and R. Ross , “Salivary Cortisol and Cortisone in the Clinical Setting,” Current Opinion in Endocrinology, Diabetes & Obesity 24, no. 3 (June 2017): 161–168. 28375882 10.1097/MED.0000000000000328

